# Multi-functional auto-fluorescent nanogels for theranostics

**DOI:** 10.1007/s13365-023-01138-y

**Published:** 2023-05-29

**Authors:** Arti Vashist, Andrea D. Raymond, Prem Chapagain, Atul Vashist, Adriana Yndart Arias, Nagesh Kolishetti, Madhavan Nair

**Affiliations:** 1 Department of Immunology and Nanomedicine, Herbert Wertheim College of Medicine, Florida International University, Miami, FL 33199 USA; 2 Institute of Neuroimmune Pharmacology, Herbert Wertheim College of Medicine, Florida International University, Miami, FL 33199 USA; 3 Department of Physics and Biomolecular Sciences Institute, Florida International University, Miami, FL 33199 USA; 4 Department of Infection & Immunology, Translational Health Science and Technology, Faridabad, Haryana 121001 India

**Keywords:** Micro/nanogel, Theranostics, Therapeutics, Imaging, Infectious diseases, Cancer, Neurological disorders

## Abstract

Here in the present article, the state of art for nanotechnology-enabled nanogel theranostics and the upcoming concepts in nanogel-based therapeutics are summarized. The benefits, innovation, and prospects of nanogel technology are also briefly presented.

## Introduction

Nanotechnology has advanced tremendously for therapeutic delivery and diagnostic application in recent years. Several nanocarriers have been tested for translation to clinics (Marrache et al. 2013). These nanocarriers include liposomes, dendrimers, polymeric nanocarriers, hydrogels, and nanogels. This review will highlight the intelligent nanocarriers, “nanogels,” used for theranostics (therapy and diagnostics) applications. Nanogels are nanomaterials in the nanometer range (10 to 100 nm) and formed via a three-dimensional network of crosslinked polymer chains or materials from gel-like structures. Nanogels have advanced properties as compared to other existing nanocarriers. The limitation associated with different nanocarriers, such as liposomes, is their stability over time and polydispersibility. Polylactic-co-glycolic acid (PLGA) and chitosan nanoparticles are accompanied by drug burst release. The inorganic particles showed toxicity issues and immune responses in clinical trials (Zhang et al. 2022). Keeping these limitations in mind, nanogels have conducted improved features. According to the standard nomenclature, IUPAC nanogel is a gel nanoparticle of any shape with a diameter of 1–100 nm (Gold et al. 1997; Kar et al. 2017). These are formed by physical or chemical cross-linked polymers. The research has expanded over the years, and nanogels have gained attention in the material science (Asadian-Birjand et al. 2012; Topuz and Uyar 2022; Vashist et al. 2017) for the theranostics application. Recently, the theranostics application of nanocarriers has been utilized to treat various diseases where the diagnostic and therapeutic are integrated with a single platform (Wang et al. 2018a). Funkhouser first defined the term “theranostics” in 2002. Since then, a lot of progress has been made in developing nanocarriers in a hybrid format to have a combinatory effect. Various nanofillers like carbon dots (Zhao et al. 2022), metal nanoparticles (Lux et al. 2015), SPIONs (Mauro et al. 2019), carbon-based materials (Wang et al. 2018b), and quantum dots (Yang et al. 2014) have been used to develop nanogels for theranostics.

The current strategy of developing bio-polymeric theranostic agents has a pronounced conviction of incorporating inherent features of non-toxicity, biodegradability, biocompatibility, and high sensitivity. Literature reveals that the nano-range is preferred for various anti-cancer therapies (Cho et al. 2020a; Pei et al. 2018) and other biomedical applications (Kaewruethai et al. 2021; Vashist et al. 2018). The focus is on developing nanogels that are easy to synthesize with low-cost materials and result in biocompatibility and biodegradability and give no toxicity to the cellular microenvironment with intrinsic photoluminescence and increased photostability. A study demonstrated nanogels based on biodegradable photoluminescent polymer templates that are derived from compatible monomers like citric acid, maleic acid, L-cysteine, and polyethylene glycol (Gyawali et al. 2018). These nanogels possess strong potential for theranostics medicine and can be used for real-time fluorescence-based imaging. They are also deployed for the cell labeling (Gyawali et al. 2018). Nanogels are better candidates selected for enhancing tumor accumulation. A recent study showed the development of fucoidan-based theranostic nanogel, which has the unique feature of recovering its photoactivity in response to intracellular redox potential when internalized into cancer cells. This study gives an excellent platform for selecting near-IR imaging and improved photothermal therapy of tumors (Cho et al. 2020b).

## Auto-fluorescent nanogels for theranostics

**Fig. 1:**
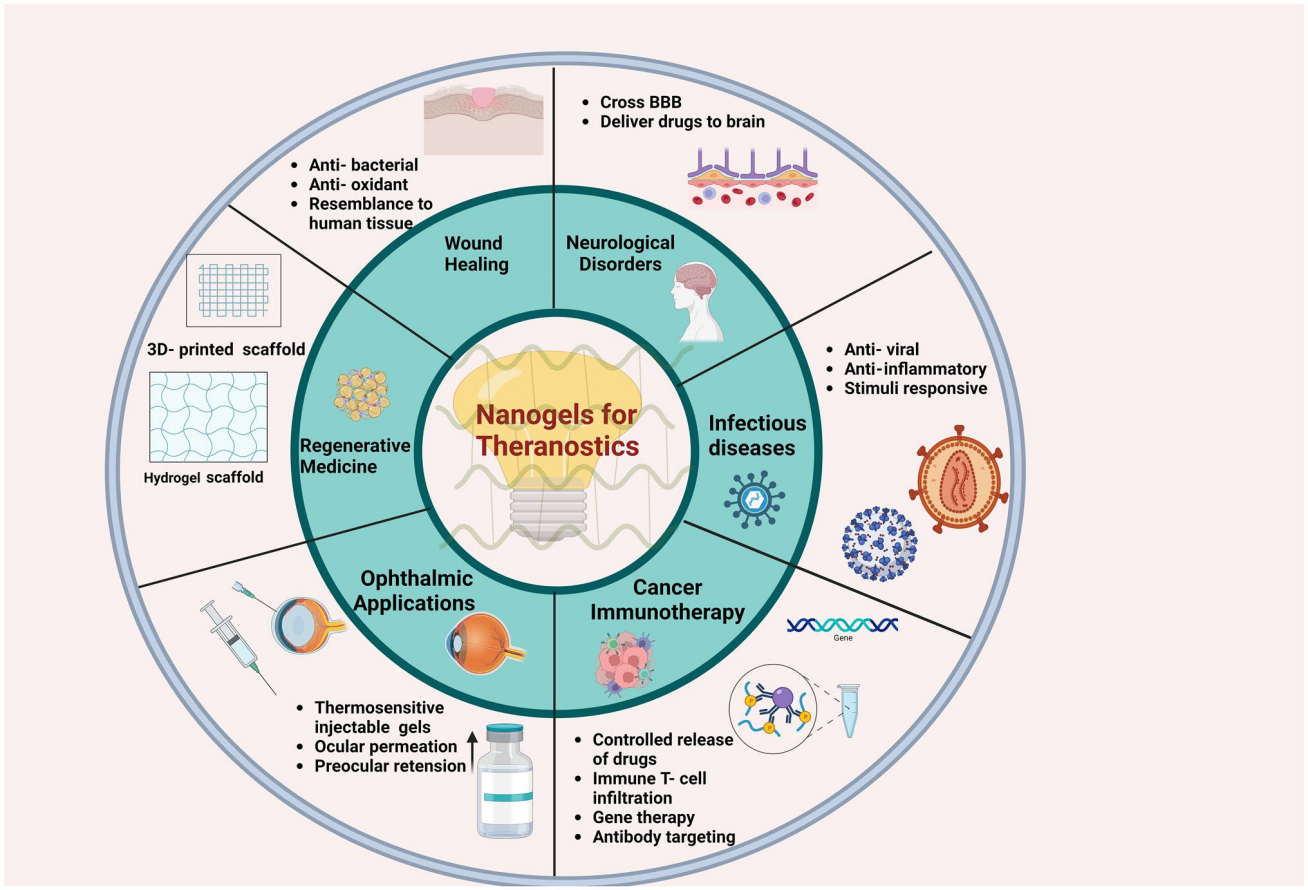
Nanogels for theranostics for various diseases. “Created with BioRender.com”

Researchers across the globe have put sincere efforts forward to develop efficient nanogel- based delivery systems to deliver hydrophobic drugs, cancer immunotherapy (Ma et al. 2022), oligonucleotides, and other bioactives across the BBB (Vinogradov et al. 2004). Recent research work on magneto-electric nanoparticles (MENP) from our group suggested that MENP-mediated drug delivery has the potential to improve the outcomes of anti-retroviral (ARV) therapy significantly and can transport neuron-resuscitating agents to cross BBB without altering the blood–brain barrier integrity (Kaushik et al. 2019, Kolishetti et al. 2022). Despite the tremendous success of the combined antiretroviral therapy (cART) in reducing the mortality rate in HIV-infected individuals, viral persistence remains a daunting challenge posing a significant barrier to HIV cure. The most critical challenge in existing therapies is that HIV infection requires lifelong adherence to daily dosing of ART, which may result in drug resistance. Besides this constant threat of the emergence of drug-resistant viral variants and overall cost compounded with adverse effects associated with ART necessitates the development of novel therapies. These therapies are aimed at targeting the integrated provirus within the host cellular DNA to eliminate the HIV viral reservoirs (Kaushik et al. 2019; Osborne et al. 2020). To address these challenges, our research group has developed and patented a very simple and stable bio-polymeric micro/nanogel system, which can be used for in vitro and in vivo theranostics applications for various diseases (Fig. 1). Owing to their novel characteristics, biopolymers, such as chitosan and hydroxyethyl cellulose (HEC), have gained huge importance for developing nanotechnology-based therapeutics and imaging tools. Bio-polymeric auto-fluorescent hydrogels in both micro- and nanoscales were developed using completely natural polymers chitosan, HEC, and sustainable resource polyol exhibiting complete biocompatibility (in the concentration range 10–100 µg/ml) tested over a wide range of host cells like astrocytes, PBMCs, and microglia (Vashist et al. 2020).

**Fig. 2:**
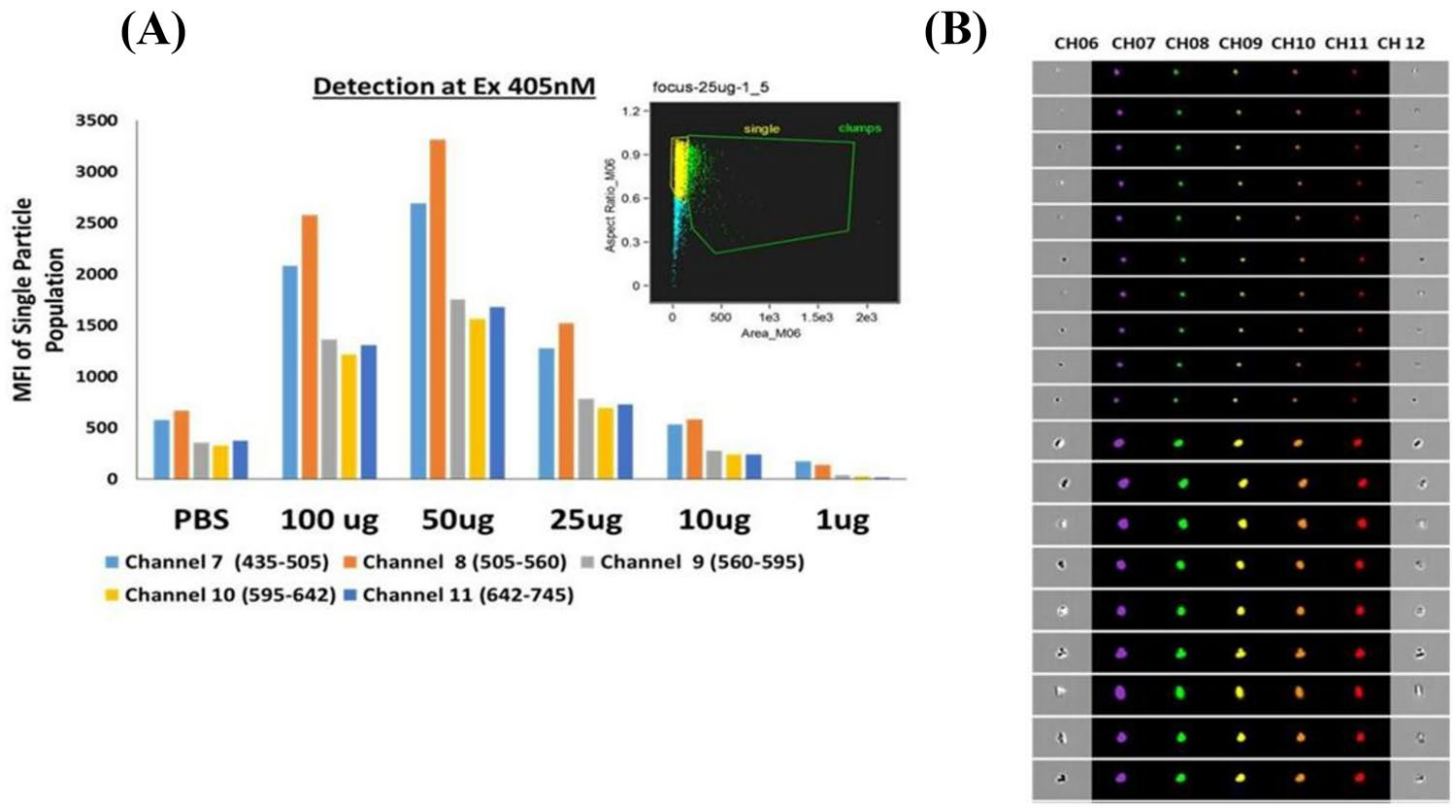
Fluorescence properties of the nanogels at various concentrations using flow cytometer. **A** The mean intensity values of the single-particle population for each channel at an excitation wavelength of 405 nm. **B** Images acquired for the presence of fluorescence for and aggregated and single particle in each channel at an excitation wavelength of 405 nm. Copyright permission from reference Vashist et al. (2020) under Creative Commons Attribution (“CC BY”) license

Moreover, their intrinsic fluorescence over a wide dynamic range of emission wavelengths (450–750 nm) and (710–810 nm) micro/nanogel particles (Fig. 2**)** obviates the requirement of an external fluorescent dye/reagent for their use in in vivo imaging-based theranostic applications (Vashist et al. 2020, 2019). The developed nanogel particles were detected with a 405-nm excitation laser and emission at 505–560 nm, demonstrating the maximum fluorescence intensity with 50 μg/ml of nanogel concentration. Our preliminary animal studies in Balb/c mice injected subcutaneously with 20 mg/kg of micro/nanogel particles revealed in vivo fluorescence of micro/nanogel particles using Ami HT-Spectral Instruments-based imaging (unpublished data not shown) underpinning them as a novel tool for in vivo imaging. Interestingly, the developed micro/nanogels significantly inhibited both viral transcription and viral release disrupting the HIV life cycle in T-cell enriched PBMCs, astrocytes, and macrophages, suggesting an inherent anti-HIV activity of synthesized micro/nanogels. Hence, the developed auto-fluorescent nanogels are anticipated to act as an anti-viral agent for HIV treatment and are proposed to be tested as a prophylactic agent against HIV in high-risk groups (Nair et al. 2022).

## Nanogel-based theranostics for CNS diseases and delivery across blood–brain barrier

**Fig. 3:**
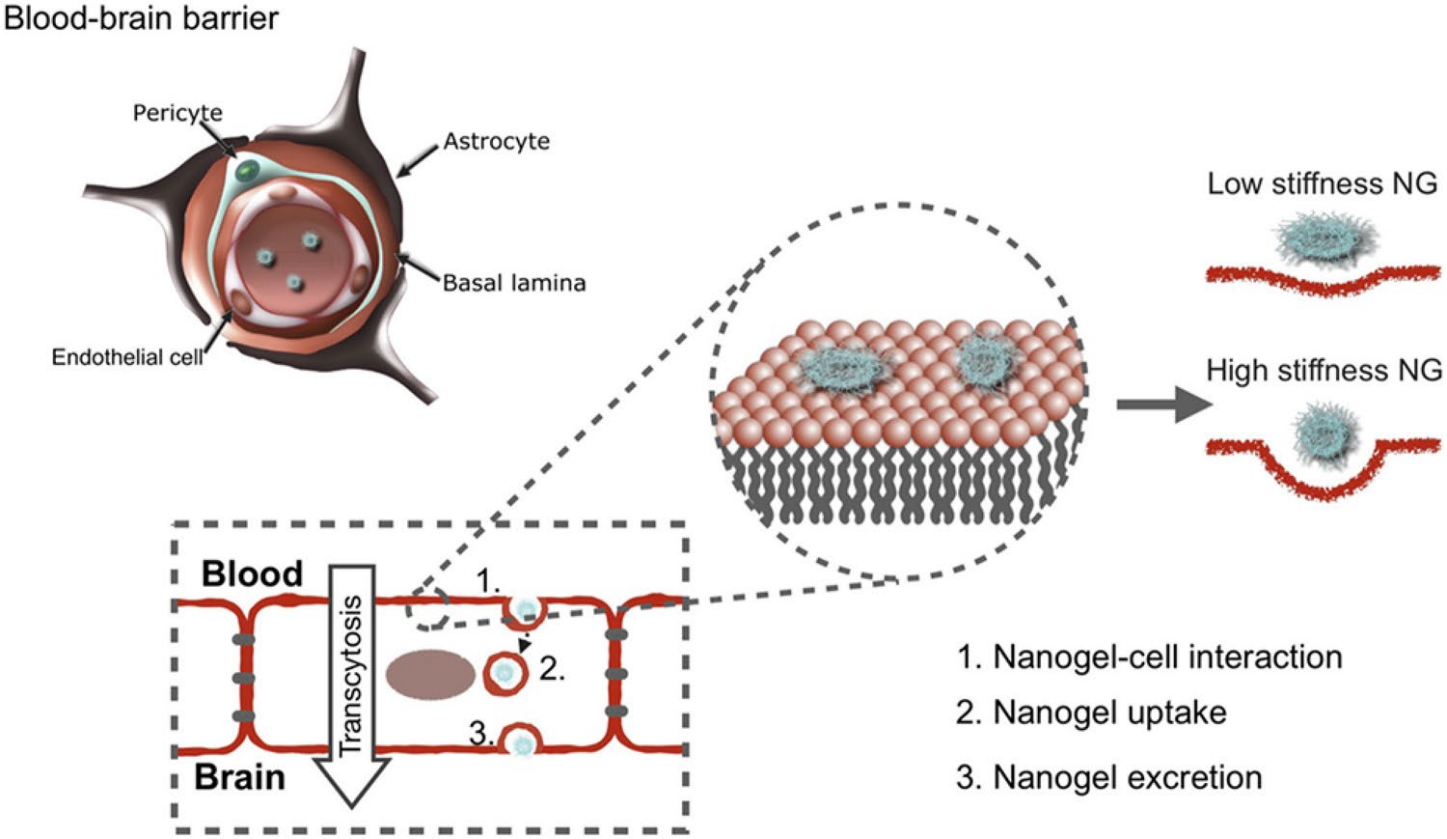
Low nanogel stiffness favors nanogel transcytosis across an in vitro blood–brain barrier. Copyright from reference Ribovski et al. 2021 Elsevier under Creative Commons CC-BY license

Nanogels hold great potential as a drug delivery system for CNS diseases. The characteristic features of nanogels which make them unique are their high stability in physiological solutions, high drug encapsulation efficiency, biodegradability, biocompatibility, core–shell structures, and high permeability and stimuli responsiveness. These properties make them better nanocarriers than other existing nanocarriers and can thus cross the blood–brain barrier to a greater extent. Nanogels are a promising carrier for achieving effective therapeutic doses across the BBB. Nanogels have been deployed for managing various brain diseases like Alzheimer’s, Parkinson’s, multiple sclerosis, brain tumor, and neuroAIDS. The presence of tough BBB and blood-cerebrospinal fluid barrier (BCSFB) makes brain drug delivery very challenging. In this regard, nanogels can be engineered so that they can be transmigrated across BBB in a non-invasive manner (Cuggino et al. 2019). It is well known that therapeutics > 500 Da cannot cross the BBB. The therapeutic has to transmigrate across the BBB to treat brain diseases effectively. In one exciting study, fluorescently labeled poly(N-isopropylmethacrylamide) (p(NIPMAM)) nanogels were tested for their stiffness and their transport. It was observed that the more densely cross-linked nanogels were uptaken by brain endothelial cells. On the contrary, the less densely cross-linked nanogels showed the highest transcytosis property (Ribovski et al. 2021). Figure 3 demonstrates that nanogels with different stiffnesses tend to interact with the apical region of polarized brain endothelial cells (Ribovski et al. 2021). Another essential aspect in these years is nasal delivery of the nanogels for enhanced brain drug delivery. Hydrogen bond-enhanced nanogel for delivery of albiflorin to treat Parkinson’s disease (PD) was reported. Glycyrrhizic acid was utilized as a hydrogen bond crosslinker, preventing the drug’s premature release. These studies demonstrate the potential of nanogels for selective brain distribution and accumulation and are promising carriers for treating PD (Chen et al. 2022). Oxytocin-loaded angiopep-2-modified chitosan nanogels have been developed to address early intervention for Alzheimer's disease (AD). These nanogels inhibit the innate inflammatory response. Oxytocin was released and explicitly bound to upregulated oxytocin receptor and thus inhibiting the microglial activation and reducing the inflammatory cytokine levels (Ye et al. 2022). Numerous other recent studies have been reported showing the potential of nanogel-based theranostics for CNS diseases and delivery across blood–brain barrier (Arjun et al. 2022; Vdovchenko 2022; Vinogradov et al. 2004).

## Future prospects and conclusion

The ideal nanocarrier can be sustained in the human body for a longer circulation time to deliver the therapeutics with minimum or no side effects. The observed non-target effects can be reduced by passive targeting (Kitayama et al. 2022) or antibody-mediated active targeting of diseased tissue or organs (Li and Qi 2021). Tagging the nanocarrier with ligands and cell-penetrating peptides may facilitate their intracellular delivery (Khalil and Harashima 2023; Liu et al. 2022). Thus, nanogels are tunable and can be functionalized to make them transmigrate across physiological barriers (Zhao et al. 2021) such as gut barrier (Lee et al. 2020), blood–brain barrier (Ribovski et al. 2021), corneal epithelial barrier (Lin et al. 2021), and skin barrier (Cuggino et al. 2019). Smart nanogel may be designed with a capacity to circulate for longer intervals with sustained release, and droplet-based microfluidic development of nanogels for controlled drug delivery can be used to achieve efficiency. Using fluorescent probes and dyes in nanocarriers for imaging purposes bears severe limitations, such as experimental artifacts, toxicity, rapid clearance, photobleaching, and low specificity. In this regard, our nanogel technology has organic biopolymers with the least toxicity and more accuracy with detectable sensitivity. There is a wide range of applications for nanogels ranging from diagnostics to therapeutics. The developed nanogels can be deployed to deliver various biologics, nucleic acids, drugs, and RNA/DNA for the treatment of brain tumors and other carcinomas in the human body, in addition to HIV therapeutics. The existing imaging technologies, such as CT, MRI, PET, and FMT, have their strength and limitations (Lim et al. 2015). The advancements in nanomaterials used for theranostics include nanofillers like CNT, graphene, superparamagnetic nanoparticles, and Au nanoparticles that enhance MRI contrast. Thus, nanogels enable superior imaging with improved diagnostic efficacy. The other important parameter for nanogel-based therapeutics is to optimize the PK and PD properties of drugs loaded inside the nanogel. Thus, clinical translation is still a big challenge. Yet, the advancement in nanogel technology and research team collaborations from multidisciplinary areas may prove to be instrumental in developing a new class of nanogels for clinical use.

## Data Availability

The datasets generated for this review are available on request to the corresponding authors.
